# Number of metastases and their response to chemotherapy impact survival of patients with isolated lung metastases from bone-derived sarcoma

**DOI:** 10.1186/s12885-021-08073-3

**Published:** 2021-04-07

**Authors:** Theresa Stork, Rebecca Boemans, Jendrik Hardes, Arne Streitbürger, Uta Dirksen, Christoph Pöttgen, Hans-Ulrich Schildhaus, Sebastian Bauer, Stéphane Collaud, Clemens Aigner

**Affiliations:** 1 Department of Thoracic Surgery, Ruhrlandklinik, University of Duisburg-Essen, Ruhrlandklinik, Tüschener Weg 40, 45239 Essen, Germany; 2 German Cancer Consortium (DKTK), Center Essen, Essen, Germany; 3 Department of Pediatrics III, University Hospital Essen, University of Duisburg-Essen, Essen, Germany; 4 Department of Tumor Orthopedics and Sarcoma Surgery, University Hospital Essen, University of Duisburg-Essen, Essen, Germany; 5 Department of Radiation Oncology, University Hospital Essen, University of Duisburg-Essen, Essen, Germany; 6 Institute of Pathology, University Hospital Essen, University of Duisburg-Essen, Essen, Germany; 7 Department of Oncology, University Hospital Essen, University of Duisburg-Essen, Essen, Germany

**Keywords:** Pulmonary metastasectomy, Bone sarcoma, Lung metastases

## Abstract

**Background:**

Pulmonary metastasectomy (PM) is an established treatment for selected patients with metastatic sarcomas. The aim of this study was to examine our institutional experience and evaluate factors predicting outcome.

**Methods:**

We retrospectively reviewed all patients undergoing PM for bone sarcoma in our center from 2001 to 2019. Survival was calculated from the date of PM. Impact on survival of clinical parameters was assessed.

**Results:**

Thirty-eight patients (27 males, 71%) were included. Histology was osteosarcoma (*n* = 20, 53%), Ewing sarcoma (*n* = 13, 34%) and chondrosarcoma (*n* = 5, 13%). Twelve patients (31.5%) had synchronous metastases, all received chemotherapy before PM. Median number of metastases was 3 (1 to 29). Twenty (53%) patients had mediastinal lymph node sampling. One patient had positive lymph nodes. Ninety-day mortality was 0%. Three and 5-year PFS were 24.5 and 21%, respectively. Three and 5-year OS were 64.5 and 38.5%, respectively. More than three metastases and progression under chemotherapy were significant independent predictors for OS.

**Conclusion:**

PM is a safe procedure and encouraging long-term outcome can be achieved. Patients with progression of pulmonary metastases under chemotherapy as well as patients with more than three metastases had significantly worse OS.

## Background

Sarcomas are rare malignancies representing a heterogeneous group of tumors. The incidence of primary bone sarcomas is approximately four to ten times lower than of soft tissue sarcomas (STS). Most frequent primary bone tumor is osteosarcoma, with an incidence of 0.3 per 100,000 per year, followed by chondrosarcoma with an incidence of 0.2 per 100,000 per year [1]. Ewing’s sarcoma is rare with an incidence of 1–3 per 1,000,000 per year [2]. Pulmonary metastases occur in up to 40% of patients with bone sarcoma [3]. Pulmonary metastasectomy (PM) is an established treatment in patients with isolated lung metastases and a potentially curative option. In patients with pulmonary metastases from bone sarcoma, 5-year survival is 20–40% [3]. Different prognostic factors such as number of metastases or disease-free interval [4–7] have been described for patients with pulmonary metastases from osteosarcoma or chondrosarcoma. Here we aim to examine our institutional experience and evaluate factors predicting outcome after PM for bone sarcomas.

## Methods

All consecutive patients who underwent PM in our institution in a curative intent for bone sarcoma from January 2001 to December 2019 were included. Data were retrospectively retrieved from the patients’ electronic documentation system and all follow-up centers. The study was approved by the institutional ethics committee (18–7943-BO).

Treatment strategy was discussed for all patients on a case-by-case basis during our dedicated multidisciplinary sarcoma tumor board. At time of primary sarcoma diagnosis all patients underwent standard x-rays, CT and/or MRT. Staging was completed with CT of the chest/abdomen, bone scintigraphy or PET/CT or PET/MRI for more recent cases. All patients with osteosarcoma, Ewing’s sarcoma and dedifferentiated chondrosarcoma had multi-agent induction and adjuvant chemotherapy. Patients with conventional chondrosarcoma did not receive chemotherapy before metastasectomy. Along the study period, regimen was not uniform for all patients. Postoperative follow up imaging for high-grade sarcoma consisted in MRI of the primary tumor’s region and a chest/abdomen CT every 3 months during the first 2 years. Imaging interval was prolonged to every 4 to 6 months during the third year and to every 6 months up to 5 years. Above 5 years, yearly imaging was discussed on a case-by-case basis. In patients with metachronous metastases decision for initial chemotherapy followed by PM or primary PM was discussed individually depending on histology and grading of primary tumor.

Indication for PM was decided on a case-by-case basis during our weekly multidisciplinary tumor board for sarcoma including radiologist, medical and radiation oncologists, orthopaedic and thoracic surgeons. All candidates for metastasectomy had to have a completely resected or locally controlled primary tumor and absence of extrathoracic disease. All pulmonary lesions have to be deemed resectable, based on preoperative CT, with sufficient postoperative lung function. Progression of metastases under chemotherapy per se was not an absolute contraindication to PM. Surgical approach was based on location, size and number of metastases. In case of two or less peripheral nodules visible on chest CT, wedge resection was performed by video assisted thoracic surgery (VATS). If nodules were multiple (three or more) or centrally located, an anterolateral thoracotomy approach was chosen. The whole lung was palpated to identify additional nodules, which were resected. Resection was preferred using cautery or laser enucleation to spare lung parenchyma. For central metastases anatomical resections were performed if required. In patients with bilateral metastases the decision to perform a one- or two-staged operation was based on fitness and comorbidities of the patient, number of metastases and preference of the patient. Mediastinal lymph node sampling was performed at surgeon’s discretion up to January 2019, while it was routinely performed by all surgeons later on.

IBM SPSS software (IBM Corp., Armonk, NY, USA) was used for statistical analysis. Kaplan-Meier curves were used to analyze survivals. Logrank test was used to compare survival curves. Survival was calculated from the day of PM until death or last follow-up (overall survival, OS) and progression at any site or last follow-up (progression-free survival, PFS). Disease free interval (DFI) was defined as the interval between primary tumor diagnosis and lung metastasis diagnosis on radiologic imaging. If pulmonary metastases were visible on initial staging imaging of the primary tumor, they were classified as synchronous metastases. The impact of the following variables – age at PM, gender, timing of metastases, progression under chemotherapy, surgical approach, size of metastases, lymph node resection and involvement, grading, number of metastases, repeat PM, unilateral/bilateral PM and histology - on survival was assessed by univariate cox regression analysis. Significant variables were computed into a multivariate analysis to assess their independent impact on survivals. A *p*-value ≤0.05 was considered significant.

## Results

Thirty-eight patients (27 males, 71%) were included. Median age at diagnosis of primary bone sarcoma was 24 years (7 to 74) and median age at first PM was 26 years (10 to 79). Histology was osteosarcoma (*n* = 20, 53%), Ewing sarcoma (*n* = 13, 34%) and chondrosarcoma (*n* = 5, 13%).

Synchronous metastases were present in twelve patients (32%). Median DFI for patients with metachronous metastases was 27.7 months (4–204). Median time from first diagnosis of pulmonary metastases to PM was 4 months (0–19). In 22 (58%) patients, chemotherapy was performed before PM. Of those patients, 12 (32%) had progression under chemotherapy. All patients with synchronous metastases received chemotherapy before PM, one patient with Ewing’s sarcoma also underwent whole lung irradiation.

**Fig. 1:**
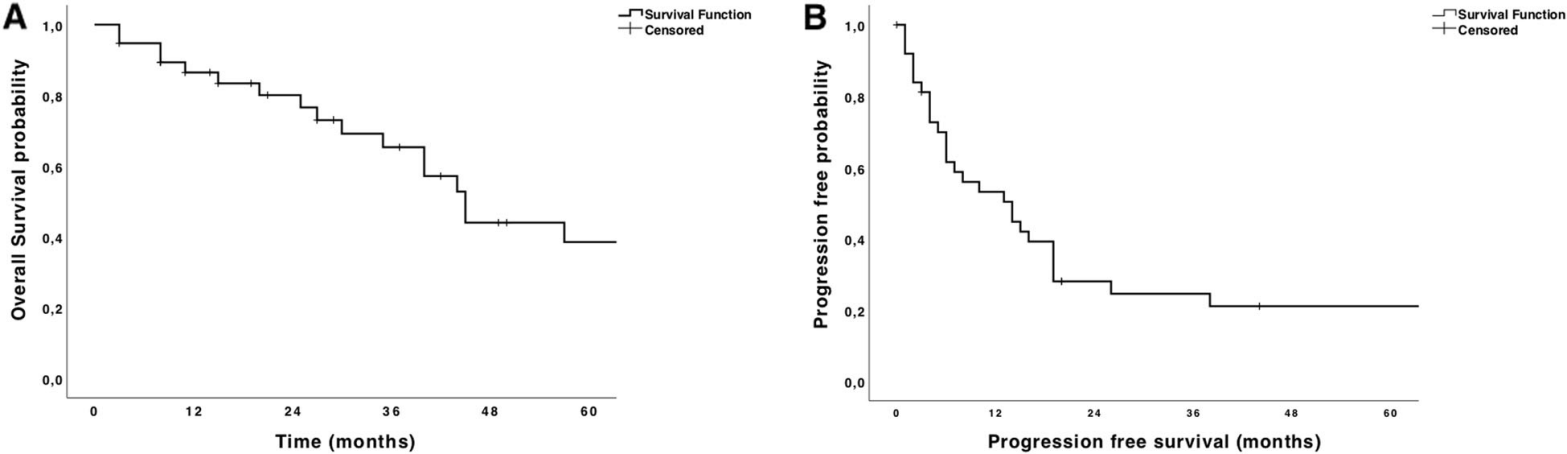
Overall (**a**) and progression-free survivals (**b**) for the whole cohort

Thoracotomy was used in the majority (*n* = 36, 95%) of patients. In 19 (50%) patients bilateral PM was performed. In 7 (37%) of those patients a simultaneous bilateral approach was chosen, 12 (63%) patients had a staged operation. The median number of metastases was 3 (1 to 29). Median size of metastases was 15 mm (0–130). Lymphadenectomy was performed in 20 (53%) patients. One single patient had positive lymph nodes. They were unexpected based on preoperative imaging and located intrapulmonary (*n* = 2) and at the level of the pulmonary ligament (*n* = 1). Nineteen (50%) patients underwent repeat PM. Postoperative complications occurred in two patients (5%). There was one patient with pneumothorax after chest tube removal requiring chest tube insertion and one patient who suffered from *C. difficile* enteritis. Ninety-day mortality was 0%. Overall and progression-free survivals are shown in Fig. 1a, b.

Median follow up was 29.5 months. Three- and 5-year OS were 65.4 and 38.5%, respectively. Three- and 5-year PFS were 24.5 and 21%, respectively. Impact of clinical variables on OS and PFS are summarized in Table 1. Disease free interval did not significantly impact OS (*p* = 0.512, HR 0.997, 95%CI 0.986–1.007) and PFS (*p* = 0.112, HR 0.991, 95%CI 0.979–1.002).

**Table 1:** Predictive factors for survival and recurrence

	Overall Survival			Progression-free Survival		
Covariates	***P*** value logrank	***P*** value cox	HR, 95%CI cox	***P*** value logrank	***P*** value cox	HR, 95%CI cox
Age at PM						
≥18 (*n* = 26)	0.615	0.620	1.279, 0.483–3.387	0.208	0.228	0.604, 0.267–1.37
< 18 (*n* = 12)						
Gender						
Male (*n* = 27)	0.671	0.675	0.81, 0.303–2.167	0.753	0.761	0.884, 0.399–1.958
Female (*n* = 11)						
Timing						
Synchronous (*n* = 12)	0.381	0.390	1.544, 0.574–4.153	0.123	0.14	1.859, 0.817–4.232
Metachronous (*n* = 25)						
Progression under CHT						
No (*n* = 12)	**0.003**	**0.011**	**0.129, 0.027–0.622**	**0.050**	**0.071**	**0.404, 0.15–1.082**
Yes (*n* = 10)						
Surgical approach						
Thoracotomy (*n* = 36)	0.272	0.478	22.451, 0.004–122,339.634	0.524	0.546	1.853, 0.251–13.692
VATS (*n* = 2)						
Metastases size						
> 15 mm (*n* = 14)	0.339	0.351	0.627, 0.235–1.674	0.725	0.734	1.145, 0.524–2.505
≤15 (*n* = 20)						
LN resected						
Yes (*n* = 20)	0.134	0.148	0.472, 0.171–1.306	0.104	0.121	0.536, 0.244–1.178
No (*n* = 16)						
LN positive						
Yes (*n* = 1)	**< 0.001**	N/A	N/A	0.072	0.130	0.174, 0.018–1.673
No (*n* = 19)						
G						
2,3 (*n* = 36)	0.6	0.606	1.474, 0.336–6.471	0.952	0.954	0.958, 0.226–4.059
1 (*n* = 2)						
N° of Metastases						
≤3 (*n* = 20)	**0.002**	**0.005**	**0.242, 0.089–0.64**	**0.001**	**0.003**	**0.291, 0.130–0.651**
> 3 (*n* = 16)						
Repeat PM						
Yes (*n* = 19)	0.573	0.579	0.766, 0.3–1.961	0.226	0.246	0.636, 0.297–1.366
No (*n* = 19)						
Unilateral/Bilateral PM						
Unilateral (*n* = 19)	0.517	0.523	0.744, 0.3–1.842	0.434	0.451	0.753, 0.36–1.573
Bilateral (*n* = 19)						
Histology (CS = 5, OST = 20, ES = 13)	0.184	0.824	1.09, 0.513–2.315	0.428	0.845	1.061, 0.587–1.915

*G* Tumor grade, *R* Completeness of resection, *LN* Lymph node, *PM* Pulmonary metastasectomy, *CS* Chrondrosarcoma, *OST* Osteosarcoma, *ES* Ewing’s sarcoma

**Fig. 2:**
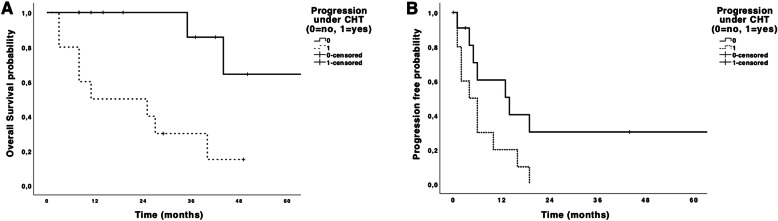
Overall (**a**) and progression-free survivals (**b**) depending on the progression of metastasis under chemotherapy

Patients with progression of metastases under chemotherapy had significantly worse OS (Fig. 2a) (*p* = 0.003) and PFS (Fig. 2b) (*p* = 0.05) compared to those without progression.

**Fig. 3:**
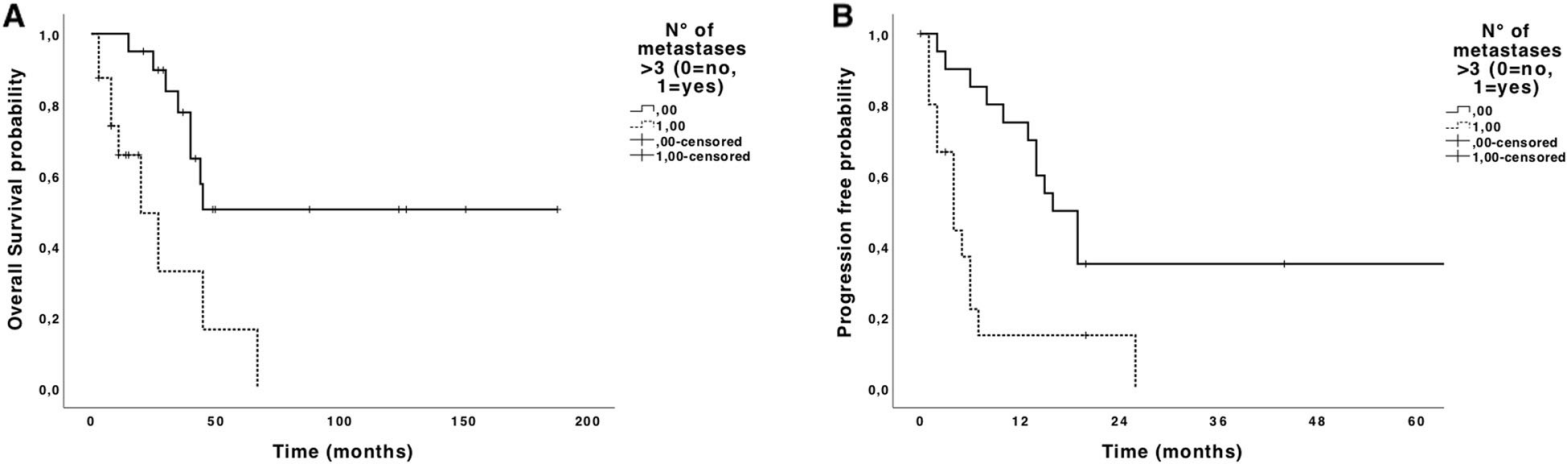
Overall (**a**) and progression-free survivals (**b**) depending on the number of metastasis

Patients with more than three lung metastases had significantly worse OS (Fig. 3a) (*p* = 0.002) and PFS (Fig. 3b) (*p* = 0.001) compared to patients with three or less metastases.

In multivariate analysis, more than three metastases variable was independently influencing OS and PFS, while progression of metastases under chemotherapy only stayed significant for predicting OS (Table 2). There was no difference in overall survival and DFS among the three different histologic entities.

**Table 2:** Multivariate analysis for overall and progression free survival

	*P*-value	Hazard ratio	95% CI
**Overall survival**			
Progression under CHT	**0.029**	6.06	1.2–30.3
N° of Metastases > 3	**0.029**	11.76	1.29–111.11
**Progression-free survival**			
Progression under CHT	0.401	1.58	0.54–4.65
N° of Metastases > 3	**0.008**	9.01	17.89–45.45

## Discussion

We retrospectively analyzed outcome and prognostic factors in patients with bone-derived sarcoma after PM. PM is widely accepted despite the lack of prospective randomized controlled trials proving its efficacy. Many retrospective studies showed a survival benefit for patients undergoing PM compared to patients undergoing non-surgical therapy [7–9]. Due to the retrospective nature of these studies, they all suffer from inherent selection bias. Indeed, the reasons not to undergo surgery are usually related to a more extended disease for the non-surgical group or severe functional limitations and comorbidities. In the study of Harting et al., 38 patients did not undergo surgery. The reasons not to undergo surgery were unresectable disease (due to a large number of metastasis or extensive mass) (*n* = 16, 42.1%), death before surgery (*n* = 6, 15.8%), patient’s refusal of surgery (*n* = 4, 10.5%), apparent resolution with chemotherapy (*n* = 2, 5.3%), extensive local recurrence before surgery (n = 1, 2.6%) or unknown (*n* = 9, 23.7%) [6].

PM was a safe procedure in our cohort. There was no major postoperative complication and no perioperative mortality. Five-year survival was 38.5% in our cohort. This is in the range of reported survivals after PM for bone derived sarcoma. A review of selected papers on PM for bone sarcoma with significant prognostic factors for OS and PFS is summarized in Table 3 [4–7, 9–11].

**Table 3:** Selected papers on PM for bone sarcoma with prognostic factors for OS and PFS

First author	Year	Histology	Number of patients with PM	5-year OS	Prognostic factors for OS	Prognostic factors for PFS
Ahmed, 2019 [4]	2008–2016	OST	88	38%	lung metastasis diagnosed during initial CHT(W) > 3 metastases (W)	lung metastasis diagnosed during initial CHT(W) > 3 metastases (W)
Briccoli, 2010 [10]	1985–2005	OST	323	37%	-	Metastasis at time of diagnosis (W) Number of PM (1 (B) vs. ≥2 (W)) DFI (24 months (B) vs. 12–24 vs. < 12(W))
Chen, 2008 [5]	1989–2007	OST	23	31%	^m^ < 5 metastases (B) ^m^ Pulmonary metastases not identified during pre- and post-operative chemotherapy (B)	< 5 metastases (B)
Harting, 2006 [6]	1980–2000	OST	93	29%	PM (B) compared to no PM (W) ^m^ DFI ≥1y (B)	–
Letourneau, 2011 [9]	1990–2006	ES	10	80%	PM (B) compared to no PM (W)	PM (B) compared to no PM (W)
Briccoli, 2004 [11]	1972–1997	ES	24	56%	PM (B) compared to no PM (W)	–
Sambri, 2019 [7]	1992–2017	CS	29	55%	^m^ PM (B) compared to no PM (W) ^m^ Bilateral metastases (W) ^m^ > 1 metastases (W)	–

*P*-values derived from logrank test if not otherwise specified. ^m^ *P*-values derived from multivariate analysis. *PM* Pulmonary metastasectomy. (B) = better and (W) = worse defined prognostic factors

In our study, seven patients (18%) lived longer than 5 years after PM. Relevant patient characteristics of these long-term survivors are summarized in Table 4.

**Table 4:** Long term survivors after PM

Patient number	Sex	Age at PM	Histology	Grading	DFI (month)	Progression of pulmonary metastases under CHT	Number of metastases (side)	Number of PM	Survival (month)
1	m	45	CS	1	161	no CHT	7 (R) + 3 (L)	2	67
2	m	21	ES	3	N/A	no CHT	N/A	3	71
3	f	12	OST	3	50	no CHT	1 (R)	1	88+
4	m	47	OST	3	30	no CHT	2 (R)	1	124+
5	m	24	OST	3	1	no progression	2 (R)	1	127+
6	m	14	OST	3	82	no progression	1 (L)	1	151+
7	m	11	OST	3	28	no CHT	1 (R)	1	188+

*M* Male, *f* Female, *N/A* Data not available, *CHT* Chemotherapy, *CS* Chondrosarcoma, *ES* Ewing’s sarcoma, *OST* Osteosarcoma, *PM* Pulmonary metastasectomy, *DFI* Disease-free interval, *R* Right, *L* Left, *+* Alive at the end of follow-up

Two patients had a total of two and three PM to achieve these results. All patients with osteosarcoma are still alive at the end of follow-up and are potentially cured from the disease. These results are encouraging since metastatic sarcoma disease is usually considered an uncurable disease.

In our study, the number of metastases was a prognostic factor for overall and progression free survival in uni- and multivariate analysis. Indeed, patients with more than 3 metastases had 12 times more risks of dying and 9 times more risks of having a progression than patients with three or less metastases. Our results confirm previous findings for both STS and bone-derived sarcomas from the literature [4, 5, 7, 12–14]. In a study investigating PM in children with osteosarcoma, better OS and PFS was also found in patients with fewer than 3 nodules in univariate analysis [4]. Results from the literature and from our cohort should prompt a more detailed description for the M stage of the TNM classification, depending on the number of metastases. According to our results, presence of distant metastases (M1) could be further divided into M1a and M1b, where M1a would be defined by the presence of one to three metastases and M1b with more than three metastases.

Use of chemotherapy is unquestionable in treatment of Ewing sarcomas and osteosarcomas. Primary tumor response to chemotherapy is known to be a strong predictor of survival and relapse in both types of sarcomas [15–17]. It was confirmed in the metastatic cohort from Harting et al., where primary tumor response to chemotherapy, as measured by tumor necrosis (≥98 vs. < 98%) was correlated with long-term survival (*p* = 0.046, 6). Five-year survival for patients with a primary tumor necrosis greater than or equal to 98% was 53.9% vs. 26.1% for those with tumor necrosis less than 98% [6]. In our cohort, patients with progression of metastases under chemotherapy had significantly worse OS and PFS compared to those without progression in univariate analysis. In the multivariate analysis, progression of metastases under chemotherapy stayed as a significant independent predictor of OS. Similar results were found by Ahmed et al., investigating the timing of occurrence of pulmonary metastases in children with osteosarcoma [4]. In a large study with 88 patients who underwent PM for osteosarcoma, the timing of appearance of metastases -at diagnosis of the primary, group 1; during initial chemotherapy, group 2; after completion of chemotherapy, group 3- were influencing outcome. Overall and event-free survival were significantly lower for patients with appearance of lung metastases during chemotherapy (8 and 6.5%, respectively) compared to both other groups. Five-year OS was 34 and 52% for groups 1 and 3, respectively. Five-year event-free survival was 18 and 25% for groups 1 and 3, respectively [4].

In a study on 23 patients with PM for osteosarcoma of the extremities, patients who developed new lung nodules under chemotherapy had a 6 times higher risk of death compared to patients who did not [5].

No evidence exists whether lymph node dissection/sampling should be performed during PM. Metastatic spread of osteosarcoma is usually hematogenic, regional lymph node involvement is rare [16]. However, it has been found to be associated with worse outcome in patients who present with regional lymph node metastases at time of diagnosis [18]. In primary lung cancer lymph node assessment is part of routine staging and lymph node involvement strongly impacts outcome [19]. Mediastinal lymph node spread in primary pulmonary sarcoma can be as high as 30% and is also impacting survival [20, 21]. In our cohort, 50% of patients had lymph node sampling at the time of PM. One single patient had pulmonary and mediastinal positive lymph nodes. He died only 98 days after PM from local recurrence to the lung, indicating that mediastinal lymph node involvement might adversely affect survival in patients with pulmonary metastatic sarcoma. Since morbidity of mediastinal lymph node sampling is low, we now perform it routinely in our center at the time of PM.

Our study suffers several limitations inherent to its retrospective nature. Data regarding primary tumor was lacking as well as reasons not to undergo PM in non-surgical candidates. Our study’s relatively small sample size is related to the rarity of bone sarcoma. It implied a long timespan for patient inclusion, during which chemotherapeutic regimen and surgical strategy evolved.

## Conclusions

The low morbidity of PM and its potential for cure in selected patients within multimodality treatment should encourage its use. The number of metastases (with a cut-off set at three) as well as the metastasis response to chemotherapy were two independent variables highly impacting OS.

## Data Availability

The datasets used and/or analysed during the current study are available from the corresponding author on reasonable request.
